# Cognitive Behavioral Therapy-Based Comprehensive Self-Management Program Improves Presenteeism in Persons with Irritable Bowel Syndrome: A Secondary Data Analysis

**DOI:** 10.3390/ijerph19053003

**Published:** 2022-03-04

**Authors:** Pei-Lin Yang, Sarah W. Matthews, Robert L. Burr, Kevin C. Cain, Pamela G. Barney, Jasmine K. Zia, Margaret H. Heitkemper, Kendra J. Kamp

**Affiliations:** 1 School of Nursing, National Defense Medical Center, Taipei 114, Taiwan; 2 Department of Child, Family, and Population Health Nursing, School of Nursing, University of Washington, Seattle, WA 98195, USA; sarahm09@uw.edu; 3 Department of Biobehavioral Nursing and Health Informatics, School of Nursing, University of Washington, Seattle, WA 98195, USA; bobburr@uw.edu (R.L.B.); pamb@uw.edu (P.G.B.); heit@uw.edu (M.H.H.); kamp@uw.edu (K.J.K.); 4 Office of Nursing Research, School of Nursing, University of Washington, Seattle, WA 98195, USA; cain@uw.edu; 5 Department of Biostatistics, University of Washington, Seattle, WA 98195, USA; 6 Department of Gastroenterology, Swedish Medical Center, Seattle, WA 98104, USA; jasmine.zia@swedish.org

**Keywords:** work productivity loss, functional gastrointestinal disorder, occupational rehabilitation

## Abstract

Individuals with irritable bowel syndrome (IBS) are more likely to miss work (absenteeism), have reduced work effectiveness (presenteeism) and experience activity impairment. This study compared the effect of a comprehensive self-management (CSM) intervention program (incorporating cognitive behavioral therapy, diet education and relaxation) versus usual care on work- and activity-impairments in adults with IBS. This secondary data analysis used daily diaries and Work Productivity and Activity Impairment in Irritable Bowel Syndrome (WPAI-IBS) questionnaire data collected at baseline, 3, 6 and 12 months post-randomization from 160 adults with IBS. Mixed-effects modeling was used to compare the effect of CSM versus usual care on work- and activity-related outcomes. The effect of CSM was shown to be superior to usual care in improving WPAI-IBS and diary-measured presenteeism, overall work productivity loss and activity impairment with sustained effects up to 12 months post-randomization (all *p* < 0.05). Moreover, the CSM intervention was found to be particularly beneficial for IBS patients with greater baseline work and activity impairments (all *p* < 0.05). The CSM intervention may bring benefits to individuals and society through improving symptoms and reducing presenteeism associated with IBS.

## 1. Introduction

Irritable bowel syndrome (IBS) is a disorder of Gut–Brain Interaction which highlights the link between emotional and cognitive brain systems with peripheral intestinal function [1]. It is estimated to affect 10~15% of the US and European populations, and it usually occurs among the working population aged 15~65 [2]. IBS has imposed a high economic burden reflected at both individual and societal levels through work productivity loss, including increased absenteeism (i.e., time missed from work) and presenteeism (i.e., reduced work effectiveness) [3]. That is, persons with IBS often not only experience gastrointestinal symptoms, such as abdominal pain/discomfort, constipation and/or diarrhea, abdominal bloating and distension [4], but also other non-gastrointestinal symptoms, such as somatic symptoms (i.e., fatigue and sleep disturbance) and/or psychological distress (i.e., anxiety and depression) [5]. These IBS symptoms can lead to impaired physical and role functioning, which, in turn, limit individual work performance and/or interfere with engagement in daily activities [6,7]. In a Swedish study, 20% of individuals with IBS reported being absent from work in the past week, with close to 90% reporting productivity impairment while at work, regardless of IBS subtypes [8].

Meta-analysis reviews support the use of cognitive behavioral therapy (CBT) and associated modalities as the best psychological strategy to treat IBS symptoms [9,10]. In CBT, thoughts (cognitions), feelings (emotions) and behaviors interact together to reduce dysfunctional cognitions about visceral sensations [11]. Additionally, CBT approaches to managing other symptoms, such as insomnia and depression, have demonstrated reductions in work- and activity-related impairment following treatment [12,13,14]. By reducing days missed from work, earlier European studies have shown that CBT is cost-effective for treating IBS [15,16,17]. However, addressing the intervention outcome as “days absent” in the majority of previous CBT-based studies on IBS populations may not be sufficient to capture the impact of IBS on work productivity. Our team developed and tested a Comprehensive Self-Management (CSM) Intervention that focused on CBT techniques and, in addition, included standard IBS diet counseling (e.g., meal timing and dietary fiber increase) and relaxation. When delivered in-person or by telephone, this program produced improvements in gastrointestinal and somatic symptoms, psychological distress, overall work productivity, and quality of life in adults with IBS compared to usual care [11,18]. However, as health-related work-productivity loss can be attributed to absenteeism and presenteeism [19], only using data on “overall work productivity” in our prior report [11] may limit our understanding of how adults with IBS were impacted by their illness, and how the CSM intervention made positive influences on these two differentiated but related phenomena of work productivity loss.

Although work and activity impairment are well-documented in IBS, few IBS interventions have addressed the impact on absenteeism, presenteeism, overall work productivity and daily activity. The few IBS interventions which address work and activity impairment are focused on pharmacological therapies; no studies have examined CBT-based therapies. For example, among IBS patients with constipation (IBS-C), treatment with linaclotide for treating constipation results in significant reductions in scores of presenteeism, overall work productivity loss and activity impairment, as well as improvement in overall work productivity through 26 weeks of treatment [20]. In the IBS literature, the impact of IBS symptoms is commonly assessed by retrospective measurement of work- and activity-related impairments. However, due to recall bias and the complexity of the outcome construct, retrospective measurement can be less accurate and often poorly correlates with daily measures [21]. Thus, the use of prospective tools, such as daily diaries, may better reflect the day-to-day impact of the treatment on behavior such as work [22]. To date, daily measures of absenteeism, presenteeism and work productivity as outcomes of CBT treatments for IBS have not been tested. The purpose of this secondary analysis was to compare the effect of the CSM intervention versus usual care on absenteeism, presenteeism, overall work productivity loss and activity impairment in adults with IBS measured both prospectively, using daily diaries, and retrospectively, using a validated questionnaire in persons with IBS.

## 2. Materials and Methods

### 2.1. Study Design and Participants

This secondary data analysis used data from a three-arm randomized controlled trial (RCT) among adults with IBS. The three arms included the Comprehensive Self-Management (CSM) Intervention delivered in-person, CSM intervention delivered over the phone and usual care (UC). Since our previous study showed no significant difference between two CSM delivery modes [11], we collapsed them together into a single CSM group for this secondary data analysis. The details of study eligibility, recruitment and other procedures have been published elsewhere [11] and registered on ClinicalTrials.gov of the US National Institute of Health (NCT00167635). Briefly, the participants were recruited through community advertisements and letters to IBS patients at a university-based gastroenterology practice. Study eligibility included a healthcare provider diagnosis of IBS and meeting the IBS Rome II Criteria, aged 18–70 years old, and no significant co-morbidity nor taking medications which could affect the outcomes of abdominal pain/discomfort or compromise their ability to complete the study.

### 2.2. Study Protocol

All eligible participants gave informed consent prior to data collection. Next, they completed baseline questionnaires, including the Work Productivity and Activity Impairment in Irritable Bowel Syndrome questionnaire. In addition, the 28-day daily diary was completed each evening during baseline data collection. Participants were randomized to CSM intervention delivered in person, CSM intervention over the telephone and usual care. Randomization was based on a computer program that used adaptive randomization [23] to ensure that the groups remained balanced on the following factors: age, sex, baseline severity of abdominal pain and predominant stool consistency. Since our previous study showed no significant difference between two CSM delivery modes [11], we collapsed them together into a single CSM group for this secondary data analysis. Participants completed a Work Productivity and Activity Impairment in Irritable Bowel Syndrome (WPAI-IBS) questionnaire and 28-day diary assessment of work productivity and activity impairment at 3, 6 and 12 months post-randomization. Among the follow-up data, the 3-month follow-up assessment was given shortly after the last CSM session. Research nurses who were blinded to the group assessments collected follow-up data. The primary study endpoints of abdominal pain and IBS-specific quality of life, as well as the secondary study points of cognitive beliefs and overall work and activity impairment for the parent study, have been reported elsewhere [11].

### 2.3. Intervention

Participants randomized to the CSM group were scheduled for nine 60-min one-on-one sections over 10 to 13 weeks [11]. The CSM sessions were delivered by trained nurse practitioners, based on an “IBS Managing Symptom Workbook”, and covered by following four main themes: education (e.g., introduction to IBS, sleep hygiene, pain management and travel), cognitive behavioral therapy–associated strategies (e.g., alternative thinking, cognitive restructuring, social support and social-skills training), IBS diet (e.g., trigger foods, dietary fiber, meal size and frequency) and relaxation (e.g., abdominal breathing and active/passive progressive relaxation) [11,24]. Participants in the CSM group received worksheets and homework assignments each week. Each session reviewed the homework from the prior week, introduced and practiced new topics and explained the homework assignments for the following week. Participants randomized to the UC group were notified that they should continue the treatments recommended by their health providers.

### 2.4. Measures

#### 2.4.1. Work Productivity and Activity Impairment in Irritable Bowel Syndrome (WPAI-IBS) Questionnaire

WPAI-IBS is a self-reported questionnaire with good psychometric properties to retrospectively assess work productivity loss and impairment in daily activities other than work over the past 7 days among individuals with IBS [25]. The WPAI-IBS contains 6 items, namely current paid employment status, hours of missed work/school due to IBS, hours of missed work/school due to other health problems, hours actually worked/attended school, the degree IBS affected productivity while working/attending school and the degree IBS affected ability of regular activities. Four variables derived from the WPAI-IBS are (1) absenteeism, percent missed work time due to IBS; (2) presenteeism, percent impairment while working/attending school; (3) overall work productivity loss, total percent work productivity loss due to absenteeism or presenteeism; and (4) activity impairment, percent impairment in the daily activities. Each variable ranges from 0% to 100%, with higher values indicating higher impairment. WPAI-IBS measures of absenteeism, presenteeism and overall work-productivity-loss data are only available for participants currently employed or are students.

#### 2.4.2. Daily Diary Measures

Daily diary measures of work productivity and activity impairment were quantified with daily diary questions over 28 consecutive days at baseline, 3, 6 and 12 months. Every evening, participants rated 4 diary items: whether went to work/school today (yes/no). If no, the reason for missing work/school, i.e., not a work/school day, cold or flu, IBS symptoms, or other. If yes, “how much did your IBS symptoms affect your productivity while you were working?” and “how much did your IBS symptoms affect your ability to carry out normal activities other than work?” Participants responded on a 4-point Likert scale from “not at all” to “very much”. Four variables derived from the daily diary were (1) absenteeism, the percentage of missed work/school days due to IBS; (2) presenteeism, the average degree IBS affected productivity on work/school days; (3) overall productivity loss, incorporating both missed workdays and impaired productivity due to IBS; and (4) IBS interference, the average degree IBS affected ability to perform regular activities. Daily diary measures of absenteeism and presenteeism are only available for participants currently employed or are students.

#### 2.4.3. Participant Demographic Characteristics

Demographic variables included self-reported age, gender, job, race and education.

### 2.5. Statistical Analysis

Data analyses were performed by using R version 3.6.1. To deal with missing data, linear mixed-effect modeling, using maximum likelihood estimation, was used [26], and participants with available data at baseline and at least one follow-up assessment time point were included. A total of 188 participants were randomized in the parent study [11], and 26 of them (13.8%) failed to provide any follow-up data on work-related data. Descriptive statistics were used to summarize demographics and outcome related to work and activity impairment. Independent *t*-test or Fisher’s Exact tests were used to examine if baseline demographics, absenteeism, presenteeism, overall work productivity and activity impairment differed between CSM and UC groups.

To address our study purpose, changes in absenteeism, presenteeism, overall work productivity and activity impairment outcomes from baseline to each follow-up time were assessed separately, using linear mixed-effects modeling for numerical outcomes, with subject as a random effect, and with treatment group, measurement time, age, gender and baseline status-related to work and activity impairment as fixed effects. The main effect of treatment group was used to examine mean changes from baseline for each outcome measure in CSM versus UC. Since treatment effectiveness might vary depending on baseline work and activity status, the baseline-by-treatment interactions were also included in our mixed-effects models [27]. Before computing the interaction term, the baseline value of absenteeism, presenteeism, overall work productivity and activity impairment was centered by subtracting the mean, so the main effect for treatment group is interpretable as being the difference between treatment groups at the mean of baseline. We also performed a 2-degree of freedom test for the joint effect of treatment and baseline-by-treatment interaction. The “lme4” package within R was used to conduct mixed-effects modeling analyses. Relationships between diary and WPAI-IBS measures for absenteeism, presenteeism, overall work productivity and activity impairment outcomes at baseline were analyzed by using Pearson correlation coefficient analysis.

We used an intent-to-treat approach in this secondary data analysis; that is, we made every effort to collect follow-up data on every randomized participant and analyzed their data based on assigned group, regardless of how many CSM sessions they received. Thus, all participants with both baseline and any follow-up data on work-related outcomes were included in the analysis [11].

## 3. Results

### 3.1. Baseline Demographics, Work Productivity and Activity-Impairment Status

Table 1 presents baseline demographic characteristics, absenteeism, presenteeism, overall work productivity and activity-impairment status. A total of 160 participants were available in this secondary analysis. The majority of participants was middle-aged, female, self-identified White, college-educated and paid employees, and reported no absenteeism related to IBS. Of the employed/student participants, 75.7% reported no hours of work missed due to IBS based on the WPAI, and 92.8% reported no days of work missed due to IBS on the diary. At baseline, neither demographics nor WPAI-IBS subscales and diary-measured work absenteeism, presenteeism, overall productivity and activity status differed statistically between treatment groups (CSM, UC) (all *p*s > 0.05).

### 3.2. Effect of CSM on Absenteeism, Presenteeism, Overall Work Productivity and Activity Impairment

**Figure 1:**
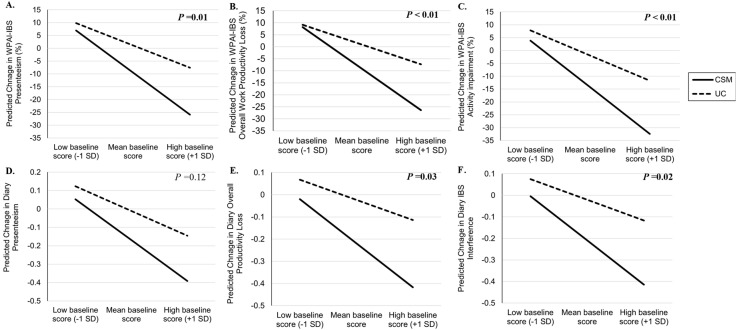
Predicted changes in WPAI-IBS and diary-measured outcomes in 3 months post-randomization; *p*-values reflect baseline-by-treatment interaction effect only. (**A**) Predicted change in WPAI-IBS presenteeism (%), (**B**) Predicted change in WPAI-IBS overall work productivity loss (%), (**C**) Predicted change in WPAI-IBS activity impairment (%), (**D**) Predicted change in diary presenteeism, (**E**) Predicted change in diary overall productivity loss, and (**F**) Predicted change in diary IBS interference.

Table 2 shows the mean changes from baseline in WPAI-IBS and diary-measured absenteeism, presenteeism, overall work productivity and activity impairment outcomes at 3, 6 and 12 months post-randomization by treatment group. For all variables, a decrease in the change from baseline (minus value) indicates improvement after treatment. As shown in the main effect of treatment comparisons between CSM and UC, the participants in the CSM group showed significantly greater reductions with small-to-medium effects in all outcomes compared to UC (Cohen’s d = −0.19~−0.32, all *p*s < 0.05), except for diary-measured absenteeism, which failed to converge due few participants reporting absenteeism. Significant small baseline-by-treatment interaction effects were also found in all outcome measures (Cohen’s d = −0.16~−0.27, all *p*s < 0.05), except diary-measured absenteeism. A time-by-treatment interaction was examined for all models but was not significant (data were shown). As shown in Figure 1, the effect of CSM compared to UC was greater among participants with more impairments at baseline.

### 3.3. Relationship between Daily Diary and WPAI-IBS Measures

Table 3 shows correlations between baseline daily diary and WPAI-IBS measures. There were significant correlations for presenteeism, overall work productivity loss and activity impairment between WPAI-IBS and diary measures (*r* = 0.44~0.51, all *p* < 0.05), but not for absenteeism (*p* = 0.75).

## 4. Discussion

To our knowledge, this is the first study to examine how a CBT-based comprehensive self-management program affects absenteeism, presenteeism, overall work productivity loss and activity impairment in the USA. Our findings show that, compared to usual care, our CBT-based CSM intervention led to greater improvements in presenteeism, work productivity loss and activity impairment measured by WPAI-IBS and daily diaries. These effects are sustained up to 12 months post-randomization (i.e., 9 months post-intervention). Short-term improvement in presenteeism, overall work productivity and activity impairments following CSM is similar to that observed with pharmacological [20,28,29] and dietary therapies [30], which report sustained effects either 4 or 26 weeks. For instance, IBS patients with diarrhea who were randomized to a low fermentable oligo-, di- and monosaccharides and polyols (FODMAP) diet showed a greater reduction in WPAI-IBS-measured presenteeism, work productivity loss and activity impairment after the 28-day intervention [30].

The mechanism of the CSM intervention leading to improvements in presenteeism, overall work and activity impairments remains to be elucidated. The positive impact of a CBT-based CSM on work and activity impairments could be through overall symptom improvement, in particular, reduction related to abdominal pain/discomfort [8,9,31], psychological distress [14] or sleep disturbance [12,13]. However, given the multicomponent nature of the CSM intervention (i.e., CBT; relaxation; IBS dietary education; and strategies to enhance self-managements, including dining and traveling), it is not possible to determine which element(s) contributed to the work-related improvements. The development of interventions specifically focused on work impairment and productivity loss in persons with IBS is challenging, since these are considered multifaceted problems correlated with IBS gastrointestinal symptom severity, as well as non-gastrointestinal symptoms, such as fatigue and anxiety [8]. The multidimensional treatment approach of the CSM intervention enabled patients to select preferred strategies for their personalized set of symptoms. Our previous findings showed that 94% of the participants in the CSM intervention still used at least six strategies, particularly relaxation, diet composition, and identifying thought distortions at the 12-month follow-up [32]. Maintaining long-term adherence to behavioral changes may explain the extensive and sustained impacts on presenteeism and activity impairment seen in our study. Our results regarding testing baseline by treatment interactions also highlight that those who experience the greatest impact of IBS on their work-life are the ones most likely to benefit. This indicates that even individuals with IBS who have severe work-related impairment can benefit from a multicomponent self-management program such as CSM.

This study extends our previous evidence by reporting that the positive impacts of the CSM intervention on overall work-productivity loss for adults with IBS are primarily through improving presenteeism, but not absenteeism. Due to a lack of model convergence, we are unable to determine if the intervention had a benefit for daily absenteeism. At baseline, only 7.2% of our employed/student participants reported absenteeism (days of work missed) on daily diaries. As such, our study may have been underpowered to detect modest changes in absenteeism with daily diaries. The rate of WPAI-IBS absenteeism in this study sample (24.3%) is comparable with the previous findings of about 20~24.3% of IBS populations reporting any hours missed from work due to IBS [8,20]. Of note, the average level of WPAI-IBS measured absenteeism in our sample (1.9~2.59%) aligns with the previous IBS studies, which showed up to 5.8% absenteeism on the WPAI-IBS [8,20,30]. Health-related work-productivity loss is attributed to not only absenteeism but also presenteeism. The rather low level of absenteeism on the WAPI-IBS in our sample verifies previous studies in IBS showing that presenteeism contributed to the majority of overall work productivity loss in IBS [8,20,28,29,30]. Working while symptomatic might explain the higher levels of presenteeism found in the US employed population-based study when those with IBS were compared to non-IBS groups [33]. Given these findings, employers may consider integrating a CBT-based intervention into regular well-being programs for employees with IBS in order to reduce indirect costs associated with presenteeism.

We also examined the correlations between daily diary and WPAI-IBS measures of work productivity and work impairment. Our correlational analysis showed, at baseline, that prospective diary and retrospective WPAI-IBS measures were significantly positively correlated; however, the diary and retrospective measures of absenteeism had low correlations. Additionally, at baseline, only 7.2% of participants reported absenteeism (days of work missed) on daily diaries, whereas 24.3% reported absenteeism on the WPAI-IBS (hours of work missed). These discrepancies in the findings could be attributed to the different measurement constructs of absenteeism between the diary and WPAI-IBS measures. That is, the WPAI-IBS captures absenteeism by using both hours missed and the entire workday, while the diary uses a single item, “missed workday”. A higher rate of absenteeism was reported on the WPAI-IBS measure than on the daily diaries, and this may suggest that missed work hours is more likely occurring than taking a whole day of leave for persons with IBS. The different types of data-collection methods may also explain these discrepancies. Prospective diaries have been preferred to retrospective reports for collection of daily health-behavior data, due to the shorter recall periods [22,34]. The WPAI can be easily integrated into busy clinical settings. However, a daily diary approach may be beneficial when clinicians/researchers are interested in the relationship between individual symptoms and absenteeism/presenteeism, as this could inform tailored interventions. As such, when assessing the impact of IBS on work-related impairment, clinical providers and researchers using daily diaries should assess hours absent. The decision to use WPAI and/or daily diaries should be driven by the clinical and research questions, as well as considerations for participant burden.

This study has several limitations. This secondary data analysis is limited to the data collected in an RCT study that did not focus on work- and activity-related impairment as the primary outcomes. Thus, employment status was not an eligibility criteria for the parent RCT study, and it was not taken into account in a randomization procedure, although the differences between the CSM and UC groups remained non-significant in daily dairy and WPAI-IBS outcomes at baseline. Given that only 7.2% of participants (n = 4 in CSM group; n = 5 in UC group) reporting days absent in our community-recruited sample and the model was unable to converge, this secondary analysis is unable to determine the effect of CSM intervention on absenteeism with daily diaries. Additional replication in more diverse IBS samples with days absent is warranted. This study showed that the effects of CSM on presenteeism, work productivity loss and activity impairment were small to medium based on statistical significance [35]; however, it remains unclear the interpretation of the magnitude of intervention based on clinical importance in IBS populations. It is suggested that additional studies examine the magnitude of intervention changes in work-related outcomes with patient satisfaction. Future research with a large sample with the most recent IBS Rome Criteria is needed to determine whether IBS symptom severity reduction leads to the positive impact of the CSM intervention on work and activity impairments.

## 5. Conclusions

In conclusion, a CBT-based CSM intervention was more effective in reducing presenteeism, overall work productivity loss and activity impairments compared to usual care for adult IBS patients, especially for those with greater baseline work and activity impairments. Therefore, individuals with IBS reporting work- and activity-related impairment can be encouraged that a comprehensive self-management not only reduces symptoms but also influences work productivity and activity impairment. It is important that future RCTs assess the impact of interventions not only on proximal outcomes of symptom management but also on more distal outcomes related to work and activity impairment. Further, we recommend clinicians and researchers assess hours absent and presenteeism, as well as prospective daily diaries instead of single-time retrospective questionnaires to evaluate the impact of IBS on work-related outcomes. Future studies are warranted to recruit larger and diverse samples to understand the CSM intervention mechanism leading to work and activity improvement.

## Figures and Tables

**Table 1:** Baseline demographics, absenteeism, presenteeism overall work productivity and activity-impairment status among individuals with IBS.

**Measure**	**Sample n = 160**					
		**CSM n = 107**			**UC n = 53**	
	**N ^1^**	**n**	**%**	**N ^1^**	**n**	**%**
Gender, females	107	94	88%	53	46	87%
Race, white	106	88	83%	53	48	91%
Education, college	107	75	70%	53	29	55%
Employed/Student	100	81	81%	48	37	77.1%
Age (mean, SD)	107	45.07	(14.09)	53	44.11	(13.95)
		**M**	**(SD)**		**M**	**(SD)**
WPAI-IBS ^3^						
Absenteeism ^2^ (%)	71	2.59	(6.30)	40	1.90	(4.89)
Presenteeism ^2^ (%)	71	26.53	(21.24)	40	22.50	(20.22)
Overall work productivity loss ^2^ (%)	71	28.00	(21.69)	40	23.19	(20.93)
Activity impairment (%)	106	30.57	(21.42)	52	33.46	(22.74)
Daily Diary ^4^						
Absenteeism ^2^ (%)	81	0.53	(2.67)	44	0.96	(2.97)
Presenteeism ^2^	85	1.56	(0.45)	46	1.58	(0.53)
Overall productivity loss	107	1.65	(0.45)	53	1.72	(0.48)
IBS interference	107	1.59	(0.44)	53	1.65	(0.48)

Note: CSM = comprehensive self-management; UC = usual care; WPAI-IBS = Work Productivity and Activity Impairment in Irritable Bowel Syndrome questionnaire. ^1^ N for available numbers of the participants. ^2^ Absenteeism, presenteeism and overall work productivity were computed for employed/student participants. ^3^ WPAI-IBS measures of absenteeism, presenteeism, overall work productivity and activity impairment ranges from 0% to 100%, with higher values indicating higher impairment. ^4^ Daily diary measures of presenteeism, overall productivity loss and IBS interferences on a 4-point scale of 1 (not at all) to 4 (very much); daily diary absenteeism ranges from 0% to 100%, with higher values indicating higher impairment.

**Table 2:** Change scores on activity-impairment and work-productivity outcomes by treatment group.

Measure	Sample n = 160								
	CSM		UC			Group Fixed Effects			
	M	(SD)	M	(SD)		Coefficient (95% CI)	*p* ^1^	Effsize	*p* ^2^
WPAI-IBS									
Absenteeism (%)					Main ^3^	−2.98 (−5.44, −0.51)	**0.02**	−0.19	**0.02**
					Inter ^4^	−3.27 (−6.50, −0.05)	**0.05**	−0.16	
3 Months	−2.34	(6.28)	4.97	(19.69)					
6 Months	−2.16	(5.86)	0.21	(4.90)					
12 Months	−1.11	(7.42)	0.16	(4.64)					
Presenteeism (%)					Main ^3^	−10.58 (−16.14, −5.05)	**<0.01**	−0.30	**<0.01**
					Inter ^4^	−7.71 (−13.53, −1.85)	**0.01**	−0.21	
3 Months	−10.63	(23.34)	3.87	(16.06)					
6 Months	−12.26	(22.28)	−0.56	(20.13)					
12 Months	−12.81	(25.10)	1.43	(21.98)					
Overall work productivity loss (%)					Main ^3^	−10.04 (−15.90, −4.21)	**<0.01**	−0.27	**<0.01**
					Inter ^4^	−9.09 (−12.16, −2.97)	**<0.01**	−0.23	
3 Months	−12.21	(24.32)	4.94	(19.66)					
6 Months	−12.13	(23.48)	−1.15	(19.93)					
12 Months	−13.42	(26.13)	−1.14	(21.17)					
Activity impairment (%)					Main ^3^	−12.33 (−17.22, −7.47)	**<0.01**	−0.31	**<0.01**
					Inter ^4^	−8.38 (−13.18, −3.56)	**<0.01**	−0.27	
3 Months	−14.62	(24.49)	−1.43	(23.54)					
6 Months	−13.07	(23.01)	−5.49	(18.15)					
12 Months	−14.59	(26.29)	−5.88	(26.99)					
Diary items									
Absenteeism (%)					The model failed to converge				
									
3 Months	−0.58	(2.83)	−1.08	(3.26)					
6 Months	0.59	(4.97)	−0.41	(2.48)					
12 Months	1.15	(5.28)	0.27	(4.36)					
Presenteeism					Main ^3^	−0.16 (−0.22, −0.05)	**<0.01**	−0.22	**<0.01**
					Inter ^4^	−0.09 (−0.20, 0.02)	0.12	−0.12	
3 Months	−0.18	(0.40)	−0.02	(0.42)					
6 Months	−0.16	(0.38)	0.01	(0.41)					
12 Months	−0.18	(0.38)	−0.02	(0.37)					
Overall productivity loss					Main ^3^	−0.20 (−0.29, −0.10)	**<0.01**	−0.32	**<0.01**
					Inter ^4^	−0.11 (−0.20, −0.01)	**0.03**	−0.18	
3 Months	−0.22	(0.40)	−0.02	(0.38)					
6 Months	−0.22	(0.37)	−0.04	(0.35)					
12 Months	−0.22	(0.40)	−0.08	(0.30)					
IBS interference					Main ^3^	−0.19 (−0.28, −0.09)	**<0.01**	−0.31	**<0.01**
					Inter ^4^	−0.11 (−0.20, −0.15)	**0.02**	−0.18	
3 Months	−0.21	(0.40)	−0.05	(0.36)					
6 Months	−0.21	(0.36)	−0.04	(0.34)					
12 Months	−0.22	(0.40)	−0.09	(0.30)					

Note: CSM = comprehensive self-management; UC = usual care; WPAI-IBS = Work Productivity and Activity Impairment in Irritable Bowel Syndrome questionnaire; 95% CI = 95% confidence interval; Effsize = Cohen’s d effect size. ^1^ *p* from mixed effects modeling analyses for testing the null hypothesis that the mean change from baseline to 3, 6 and 12 months is the same in both CSM and UC groups, based on a model that includes subject as a random effect and treatment group, measurement time, gender, age, baseline of the outcome and treatment group by baseline as fixed effects. ^2^ *p* 2-degree of freedom test of the joint effect of treatment and baseline-by-treatment interaction. ^3^ Main: the main effect regarding treatment comparison between CSM and UC. ^4^ Inter: baseline-by-treatment interaction effect. Bold values denote the significance at the *p* < 0.05 level.

**Table 3:** Correlations of work productivity and activity impairments outcomes between daily diary and WPAI-IBS measures.

Measure		Correlations	
Daily Diary	WPAI-IBS	*r*^1^ (95% CI)	*p*
Absenteeism	Absenteeism	0.03 (−0.13, 0.18)	0.75
Presenteeism	Presenteeism	0.51 (0.39, 0.62)	<0.01
Overall productivity loss	Overall work productivity loss	0.45 (0.32, 0.57)	<0.01
IBS interference	Activity impairment	0.44 (0.31, 0.56)	<0.01

Note: WPAI-IBS = Work Productivity and Activity Impairment in Irritable Bowel Syndrome questionnaire; CI = confidence interval. ^1^ Pearson r coefficients are reported.

## Data Availability

The data and materials included in this secondary data analysis are available from the senior author (M.M.H.), upon a reasonable request.
